# Detection of the Potential Inactivation of Tetrodotoxin by Lactic Acid Bacterial Exopolysaccharide

**DOI:** 10.3390/toxins10070288

**Published:** 2018-07-12

**Authors:** Nguyen Hoang Khue Tu, Nghe Van Dat, Le Van Canh, Doan Thi Thanh Vinh

**Affiliations:** Department of Biotechnology, School of Biotechnology, Hochiminh City International University, Vietnam National University-Hochiminh City, Quarter 6, Linh Trung Ward, Thu Duc District, Hochiminh 700000, Vietnam; nghevandat@gmail.com (N.V.D.); vancanh105@gmail.com (L.V.C.); dttvinh@hcmiu.edu.vn (D.T.T.V.)

**Keywords:** lactic acid bacteria, tetrodotoxin, exopolysaccharide, detoxification, cuprous oxide

## Abstract

Screening for compounds that can neutralize the toxicity of tetrodotoxin (TTX) or reduce its negative effects is necessary. Our study tested the TTX detoxification capacity of exopolysaccharide (EPS) extracted from lactic acid bacteria. EPS of *Leuconostoc mesenteroides* N3 isolated from the Vung Tau sea (Vietnam), *Lactobacillus plantarum* PN05, and *Lactobacillus rhamnosus* PN04 were used in the study. To more completely evaluate the importance of EPS in detoxification, EPS samples of *Leuconostoc mesenteroides* N3, *Lactobacillus plantarum* PN05 and *Lactobacillus rhamnosus* PN04 were also tested. The majority of EPS of these bacteria contained glucose; this was observed using thin layer chromatography (TLC) and high-performance liquid chromatography (HPLC) analysis. As observed with FTIR analysis, only EPS of *Lactobacillus plantarum* PN05 contained methyl groups. The results indicated that detoxification of TTX in mice could be obtained at an optimal dose of 248 µg EPS from *Leuconostoc mesenteroides* incubated with 54 µg cuprous oxide for 40 min or 148 µg EPS *Lactobacillus rhamnosus* incubated with 55 µg cuprous oxide for 40 min, while EPS from *Lactobacillus plantarum* showed TTX detoxification capacity without cuprous oxide combination. Consequently, EPS from *Lactobacillus plantarum* PN05 can be used in TTX prevention. This is the first report on the importance of lactic acid bacteria in TTX detoxification.

## 1. Introduction

Up to now, an antidote for tetrodotoxin (TTX) poisoning has not been developed. It is necessary to find an effective method to minimize the risks associated with clinical trials of TTX.

Many researchers proved that TTX was produced by symbiotic bacteria, and then accumulated in different organs of puffer fishes [1,2,3,4]. TTX was also found commonly in various types of animal including the California newt [5], the goby [6], the gastropod mollusks [7], the xanthid crab [8], and the blue-ringed octopus [9]. TTX inhibits the initiation and conduction of potential action by selectively blocking sodium channels on the nerve and muscle membrane at extremely low concentrations [10]. Furthermore, TTX-containing puffer fish meat was traditionally fermented before use in Japan [11].

Lactic acid bacteria (LAB) are used as probiotics. Lactic acid bacteria can be divided into homopolysaccharides or heteropolysaccharides to protect themselves from unfavorable culture conditions such as desiccation, osmotic stress, antibiotics or toxic compounds, predation by protozoans, phagocytosis and phage attack [12,13]. There are varieties of heteropolysaccharide categorized according to their structure, composition, molecular mass, and functionalities. Exopolysaccharide (EPS) production from LAB is strongly influenced by culture conditions, resulting in different structures and functions [14]. The EPS from *Streptococcus thermophilus* CRL 1190 was found to be effective for preventing chronic gastritis [15]. The EPS of *Lactobacillus paracasei* subsp. *paracasei* NTU 101 and *Lactobacillus plantarum* NTU 102 demonstrated potential antioxidant properties [16]. EPS can also interact with copper forming a complex structure and conferring specific activities in EPS [17].

As the above stated above, the study investigated TTX detoxification using *Lactobacilli* exopolysaccharide. 

## 2. Results

### 2.1. Identification of Leuconostoc mesenteroides N3

After checking the bacterium showing short chain of coccal shape under the microscope, bacterium was identified by biochemical tests and 16S rRNA sequencing analysis. As a result, the bacterium was found to be a facultative anaerobe. It can utilize and produce acid by fermenting d-glucose, mannitol, rhamnose, d-sorbitol, mannose, d-galactose, d-fructose, and 10% lactose. In addition, this bacterium appeared positive for indole, urease, amylolysis, phenylalanine deaminase, utilization of malonate, and methyl red tests, but negative for sucrose, maltose and l-arabinose, Voges–Proskauer reaction, gelatin liquefaction, tryptophan deaminase, ornithine decarboxylase, phenylalanine deaminase, and arginine decarboxylase. The partial 16S rRNA sequence was 425 bp deposited in DDBJ (accession number: LC066674). The partial 16S rRNA gene sequence was analyzed using NCBI BLAST, showing 99% of the homology to *Leuconostoc mesenteroides*. The isolated strain was identified as *Leuconostoc mesenteroides* N3.

### 2.2. EPS Isolation

The EPS yield of *Leuconostoc mesenteroides*, *Lactobacillus plantarum,* and *Lactobacillus rhamnosus* was 140 mg, 150 mg, and 140 mg per 10^6^ cfu/mL, respectively. However, the EPS solubility in water of isolated *Leuconostoc mesenteroides*, *Lactobacillus plantarum,* and *Lactobacillus rhamnosus* was 9.88%, 67.73%, and 9.26%, respectively (Table 1). 

### 2.3. Monomer Detection

**Figure 1:**
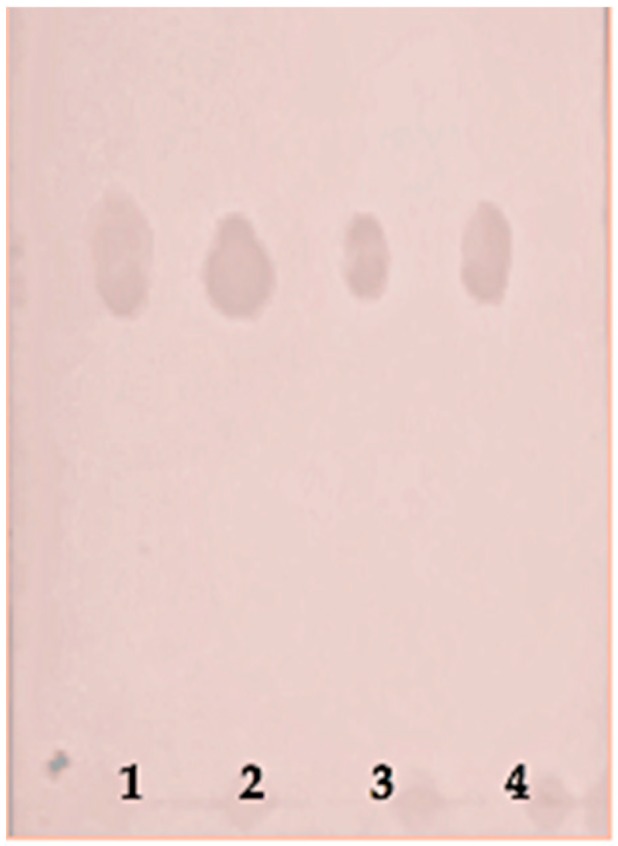
The hydrolyzed exopolysaccharide (EPS) of *Leuconostoc mesenteroides* (1), *Lactobacillus plantarum* (2), and *Lactobacillus rhamnosus* (3) were detected by thin layer chromatography (TLC). Standard glucose (4).

By thin layer chromatography (TLC) analysis, the EPS of *Leuconostoc mesenteroides*, *Lactobacillus plantarum*, and *Lactobacillus rhamnosus* was constituted mostly by glucose. The R_f_ value of standard d-glucose was 0.73, similarly to the spots of monomer from the EPS of *Leuconostoc mesenteroides*, *Lactobacillus plantarum*, and *Lactobacillus rhamnosus* (Figure 1).

The monomer detection was also confirmed by high-performance liquid chromatography (HPLC). The results of the HPLC showed that EPS from *Leuconostoc mesenteroides*, *Lactobacillus plantarum,* and *Lactobacillus rhamnosus* consisted of d-glucose and one unknown monomer. All hydrolyzed peaks appear at the same retention time of standard d-glucose (Table 2). Moreover, the ratio between d-glucose and the unknown monomer was highest in the EPS of *Lactobacillus plantarum*, then the EPS of *Leuconostoc mesenteroides*, and finally lowest in the EPS of *Lactobacillus rhamnosus*. These differences could result in a different TTX detoxification capacity.

### 2.4. Functional Group Detection

FTIR was performed for more clarification on the structure of EPS and summarized in Table 3. The polymers had group frequencies including OH, CH_2_, CH, C–C, C=O, C–O at 3425 cm^−1^, 2934 cm^−1^, 1457 cm^−1^, 1224 cm^−1^, 1645 cm^−1^, and 1056 cm^−1^, respectively. Significantly, the EPS of *Lactobacillus plantarum* contained a different frequency at 1380.7 cm^−1^ while 1456.3 cm^−1^ and 1457.4 cm^−1^ in the EPS of *Lactobacillus rhamnosus* and *Leuconostoc mesenteroides,* respectively. It was revealed that the EPS of *Lactobacillus plantarum* contained a dimethyl group in its monomer. This difference could make the EPS of *Lactobacillus plantarum* have a different activity in comparison with that of the EPS of *Lactobacillus rhamnosus* and *Leuconostoc mesenteroides*.

### 2.5. Mice Assay for TTX Detoxification Capacity of EPS

TTX was incubated with the EPS of *Leuconostoc mesenteroides*, *Lactobacillus plantarum*, and *Lactobacillus rhamnosus* together with cuprous oxide at room temperature for 20 to 60 min. On the basis of the lethal dose (0.22 µg TTX/mouse unit) that caused 100% of mice to die within 30 min, the TTX dose applied to the mice depending on their weight is presented in Table 4. 

### 2.6. TTX Detoxification Capacity of EPS of Lactobacillus rhamnosus

**Figure 2:**
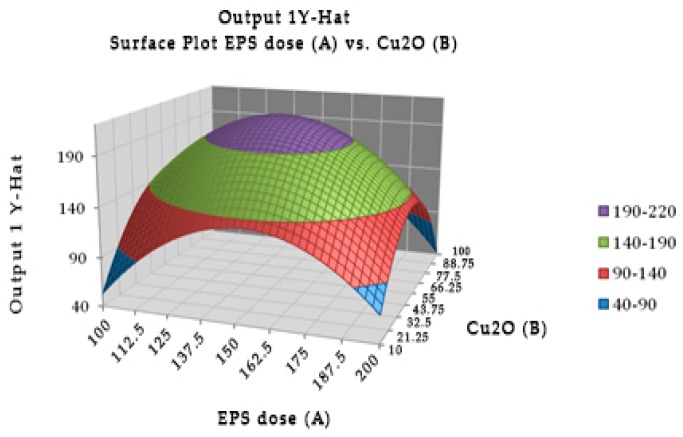
Surface and contour plot of combination of EPS of *Lactobacillus rhamnosus*, cuprous oxide, and time treatment for TTX detoxification capacity on mice.

Experimentally, the optimal combination of EPS of *Lactobacillus rhamnosus* (147.946 µg) with cuprous oxide (55.325 µg) incubated for 40.415 min showed the highest TTX detoxification capacity, resulting in all mice recovering after injection (Figure 2). EPS or cuprous oxide alone did not help mice recover from TTX. There was no significant difference in the incubation time from 20 to 60 min in the TTX detoxification capacity. Table 5 showed the reduction percentage of TTX incubated with EPS (28.99%) or both EPS and cuprous oxide (37.81%). The interaction between the EPS of *Lactobacillus rhamnosus* and cuprous oxide in TTX detoxification may occur in a shorter time, less than 20 min.

### 2.7. TTX Detoxification Capacity of EPS of Lactobacillus plantarum

**Figure 3:**
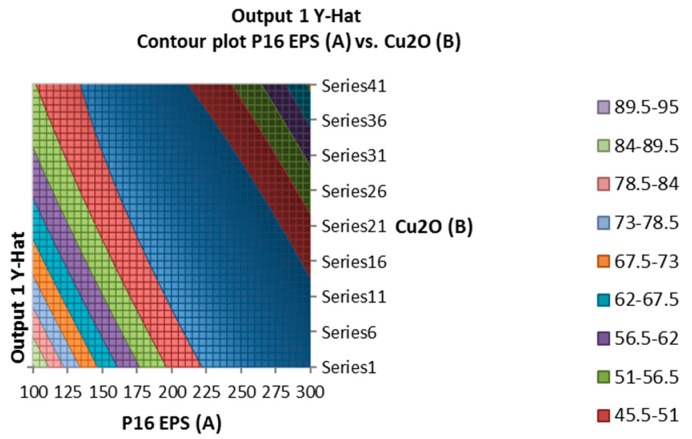
Surface and contour plot of combination of EPS of *Lactobacillus plantarum*, Cu_2_O, and time treatment for TTX detoxification capacity on mice.

In the Figure 3, the mixture of the EPS of *Lactobacillus plantarum* and cuprous oxide did not show any TTX detoxification in mice. However, the EPS of *Lactobacillus plantarum* showed TTX detoxification capacity in mice. Actually, EPS showed the higher reduction percentage of TTX than EPS incubated with cuprous oxide when analyzed by HPLC (Table 5).

### 2.8. TTX Detoxification Capacity of EPS of Leuconostoc mesenteroides

**Figure 4:**
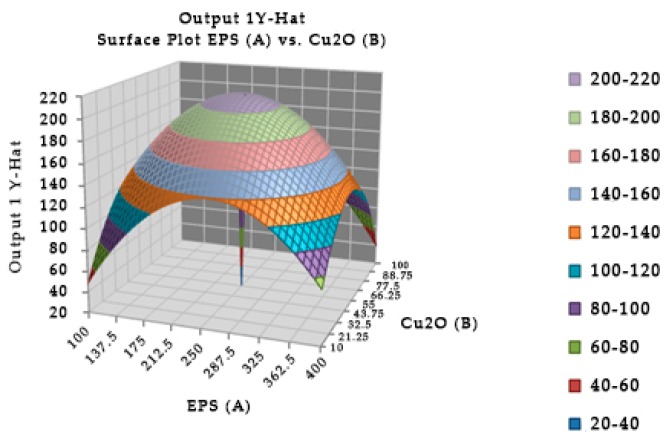
Surface and contour plot of combination of EPS of *Leuconostoc mesenteroides*, cuprous oxide, and time treatment for TTX detoxification capacity on mice.

The combination of the EPS of *Leuconostoc mesenteroides* and cuprous oxide significantly affected the survival in mice (Figure 4). In experiment, the optimal incubation time was 40.04 min, the optimal EPS dose was 248.449 µg, and cuprous oxide dose was 54.204 µg for mice survival after injection. In the study, the incubation times of EPS and cuprous oxide were changed from 20 to 60 min which insignificantly affected the survival in mice. Table 5 showed that the reduction percentage of TTX incubated with EPS (5.96%) was lower than the EPS incubated with cuprous oxide (37.54%). The interaction between the EPS of *Leuconostoc mesenteroides* and cuprous oxide in TTX detoxification may occur in a shorter time, less than 20 min.

## 3. Discussion

*Lactobacillus plantarum* PN05, *Leuconostoc mesenteroides* N3, and *Lactobacillus rhamnosus* PN04 were chosen to clarify TTX detoxification. EPS is a countering compound produced by lactic acid bacteria in response to disadvantageous conditions, hypothesized as a TTX detoxification compound in this study. Different bacteria may produce EPS differently in response to hard conditions, resulting in different activities [18]. EPS may directly bind and reduce the toxicity of antibiotic or prevent antibiotic binding to active sites. Other studies revealed that TTX can be removed from puffer fish meat by traditional fermentation in which lactic acid bacteria play the main roles [11]. According to researched results, EPS was chosen and scanned for its TTX detoxification capacity. 

*Lactobacillus plantarum*, *Leuconostoc mesenteroides,* and *Lactobacillus rhamnosus* had an equal productivity of EPS, but varied their solubility in water largely because of the difference of functional groups detected by FTIR, leading to the different interaction with water and other compounds. 

From the Box–Behnken design, the experimentally optimal results indicated that the EPS of *Leuconostoc mesenteroides* and *Lactobacillus rhamnosus* can detoxify TTX and help mice survive when combined with cuprous oxide, while the EPS of *Lactobacillus plantarum* alone showed TTX detoxification capacity. This variation may be due to the different structure of EPS of the three strains. The study suggested that the EPS of *Lactobacillus plantarum* alone could be used in prevent TTX contamination. 

**Figure 5:**
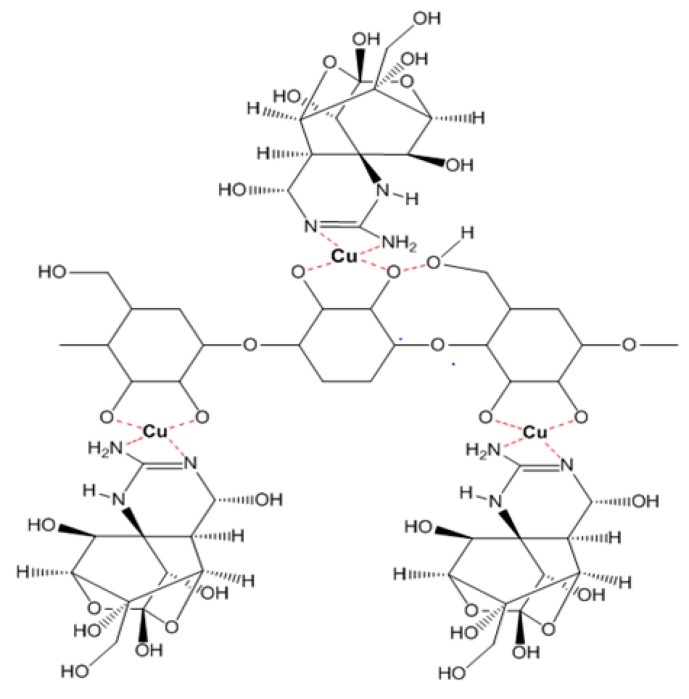
The interaction model of TTX and EPS from *Leuconostoc mesenteroides N3* and *Lactobacillus rhamnosus PN04* through Cu bridges.

**Figure 6:**
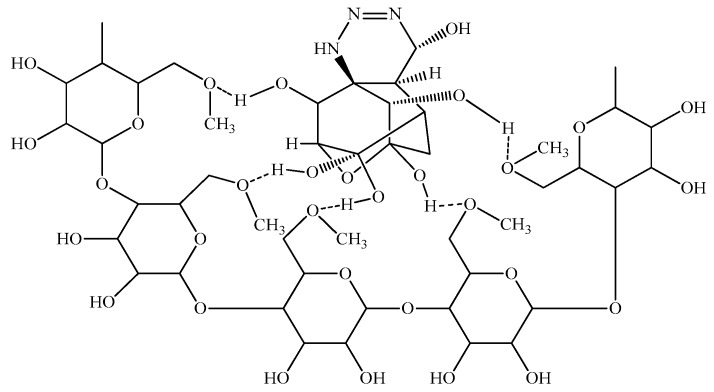
The interaction model of TTX and EPS isolated from *Lactobacillus plantarum PN05*.

In the cases of the EPS of *Leuconostoc mesenteroides* and *Lactobacillus rhamnosus*, the EPS showed their TTX detoxification capacity when combined with copper ion. EPS is a long chain polymer while tetrodotoxin contains many strong functional groups in a compact structure. In the presence of copper, both EPS structures of *Leuconostoc mesenteroides* and *Lactobacillus rhamnosus* contain functional groups (O–H, N–H) that interact with copper forming a complexly compact structure that can bind to TTX through many hydrogen bonds (Figure 5), then TTX was not enough amount to cause TTX toxicity in mice. According to the FTIR result, the EPS of *Lactobacillus plantarum* was methylated. This group makes the electric density in oxygen become more negative in charge allowing them to interact with hydrogen of the hydroxyl groups of TTX (Figure 6). These interactions might happen faster than the interaction of copper with nitrogen in nitro moiety of TTX leading to TTX detoxification without the presence of copper. Further investigations must be conducted to optimize the way EPS originated in many sources in TTX detoxification.

## 4. Conclusions

EPS from *Leuconostoc mesenteroides N3* and *Lactobacillus rhamnosus PN04* combined with cuprous oxide can detoxify TTX in mouse. However, because cuprous oxide is toxic, these combinations should be considered well. Fortunately, EPS from *Lactobacillus plantarum PN05* alone showed TTX detoxification capacity in mice. Therefore, the study may bring out the potential way in TTX prevention.

## 5. Materials and Methods

### 5.1. Isolation and Identification of Leuconostoc mesenteroides N3

The seawater samples were collected at different places in the Vung Tau sea in Vietnam. These samples were handled in sterile condition. The isolation for lactic acid bacteria was done according to Schillinger [19] and Rodriguez et al. [15]. The media including the de Man–Rogosa–Sharpe (MRS) agar or broth (Merck, Darmstadt, Germany) were used. Briefly, a 10 g of sample was mixed with 90 mL saline solution (0.9%) to get a ratio of 1/10. The solutions were shaken for 10 min to homogenize. A volume of 1 mL was plated on MRS agar and incubated in CO_2_ (5%) conditions at 45 °C for 48 h. About ten colonies were grown at the incubated condition. All colonies were taken for identification. The isolated colonies were tested by microscopic examination with gram stain and catalase production. The pure colonies were also characterized using an uronic acid test. Then, the strains were identified by biochemical characterization based on the ability of the isolates to utilize different carbon sources, which were determined by API CHL 50 (bioMerieux, Lyon, France) and 16S rRNA sequencing analysis. The forward primer was f1 (5′-GCAAGTCGAACGCACAGCGA-3′) and the reverse primer was f2 (5′-CACGTATTTAGCCGTCCCTTTC-3′). The PCR reaction was performed as follows: 95 °C for 5 min; 30 cycles of 95 °C for 20 s, 50 °C for 20 s, and 72 °C for 3 min; and a final extension at 72 °C for 10 min. The PCR product was stored at 4 °C. The purified PCR product was then sequenced. The homology comparison of 16S rRNA gene sequence with the others was performed using BLAST (NCBI).

### 5.2. Cultivation of Lactic Acid Bacteria for EPS Isolation

Lactic acid bacteria strains used in this study including *Leuconostoc mesenteroides* N3, *Lactobacillus plantarum* PN05 isolated from *Coriandrum sativum* [20], and *Lactobacillus rhamnosus* PN04 isolated from *Hottuynia cordata* Thunb [21]. All these strains were cultured in MRS broth at room temperature for 48 h.

### 5.3. Mice

Mice (20 ± 1 g) selected for this experiment were male, provided by the cell reprograming laboratory, Hochiminh City International University (HCM-IU), Vietnam National University—Hochiminh city. All the procedures were done according to the rules of HCM-IU (Ethical approval code: 302/QD-DHQT-QLKH; Renewed date of approval: 2 July 2018). 

### 5.4. Isolation of EPS

All supernatant was harvested after centrifugation at 10,000 rpm, 4 °C for 30 min. The procedure was done according to Wei’s group [22]. The dried EPS was used for quantification, further purification for structure determination, and testing for TTX neutralization. 

### 5.5. EPS Determination

The EPS concentration was determined by the phenol-sulphuric acid method [23]. The absorbance was measured at 490 nm. The concentration of EPS was determined in triplicate. Distilled water was used as blank. The EPS content of each sample was calculated based on the standard curve.

### 5.6. Molecular Weight Determination

The extracted EPS was purified according to the method [24]. The precipitated EPS was dissolved in water, dialyzed in water, and lyophilized. The UV spectrum of the purified EPS was investigated for the presence of protein or nucleic acid. The molecular weight of EPS was determined by performing gel permeation chromatography (GPC). The EPS was eluted with 0.1 NaNO_3_ at a flow rate of 0.6 mL/min. Dextran was used as standard to estimate the molecular weight of EPS [25].

### 5.7. Monomer Detection

Monomers of EPS were preliminary detected by thin layer chromatography [26]. After that, high performance liquid chromatography was performed to thoroughly detect for monomers. The hydrolyzed EPS was dissolved in Milli-Q water and applied into the monosaccharide Ca^2+^ column. Milli-Q water was used as mobile phase. The flow rate was 0.6 mL/min. The monosaccharides such as d-glucose, d-galactose, fructose, d-mannose, d-xylose, and l-rhamnose were used as standards. Refractive Index Detector (RID) was used in the study [26].

### 5.8. FT-IR

FTIR was performed to detect functional groups or chemical bonds in the compound according to the previous method [27]. To detect functional group present in the EPS, FTIR was carried out by micro-KBr pellet technique. Lyophilized EPS powder was ground with potassium bromide powder finely. A spectrum was recorded using FTIR spectrophotometer (Nicolet-5700 Thermo Electron Corporation, Madison, WI, USA) in the frequency range of 400–4000 cm^−1^. Potassium bromide was used as a background reference.

### 5.9. Test for TTX Neutralization Capacity of EPS

TTX in a range of 0.1–0.5 µg was screened on mice via intraperitoneal injection to determine minimal dose at which 100% mice died within 30 min. After determination of exact lethal dose, TTX was combined with EPS of *Leuconostoc mesenteroides*, *Lactobacillus plantarum,* and *Lactobacillus rhamnosus* with and without cuprous oxide. The mixtures were incubated for 20–60 min, then intraperitoneally injected in mice. The symptoms and time of death of injected mice were recorded carefully. In parallel, we intraperitoneally injected mice with TTX, and then injected EPS or EPS combined with cuprous oxide. All the symptoms and time of death were then recorded. Box–Behnken design was used to set up the doses for TTX detoxification in mice.

### 5.10. Statistical Analysis and Box–Behnken Design

In order to maximize the TTX detoxification capacity of EPS, the different concentrations of EPS and Cu_2_O as well as incubated time were screened. A set of factors was studied at three levels (−1, 0, +1) those are standing for the lower extreme, central point and the higher extreme of each factor in the design respectively (Table 6, Table 7 and Table 8). These factors were symbolized as A, B, and C, respectively.

The experimental matrix was designed by using the Design-Expert@ version 8.06 (Stat-Ease Inc, East Hennepin Avenue, Minneapolis, MN, USA) design was arranged in Table 9. 

On the basis of Box–Behnken design, the experiments for determination of optimal combination of EPS and cuprous oxide in a suitable incubation time were done. All the results were statistically analyzed.

## Figures and Tables

**Table 1:** Characteristics of exopolysaccharide (EPS) obtained from lactic acid bacteria.

Selected LAB	Crude EPS (mg/0.5 L Culture)	Water Solubilizing EPS (mg/0.5 L Culture)	Percentage (%)	Molecular Weight (Da)
*Leuconostoc mesenteroides*	140	13.83	9.88	3.8 × 10^4^
*Lactobacillus plantarum*	150	101.6	67.73	4 × 10^4^
*Lactobacillus rhamnosus*	140	12.96	9.26	4.4 × 10^4^

**Table 2:** The retention time and peak area of monomer revealed by high-performance liquid chromatography (HPLC).

Hydrolyzed EPS from Selected Lactic Acid Bacteria (LAB)	Peak Area	Retention Time (min)
*Leuconostoc mesenteroides*	143,843	13.133
	87,661	15.599
*Lactobacillus plantarum*	82,310	13.125
	53,584	15.575
*Lactobacillus rhamnosus*	120,313	13.152
	34,456	15.620
Standard d-glucose	579,056	15.512

**Table 3:** The Wavenumber (cm^−1^) corresponding to chemical bonds from FTIR spectrum.

EPS from Selected LAB	Wavenumber (cm^−1^)							
	OH	C–H	C=O	N–H	C–H	C–C	C–O	C–H
*Leuconostoc mesenteroides*	3427.5	2934.4	1645.1	1542.5	1457.4	1224.6	1056.2	813.4
*Lactobacillus plantarum*	3438.2	2936.8	1654.2	1550.9	1380.7	1223.4	1052.4	814.8
*Lactobacillus rhamnosus*	3421.9	2934.1	1652.1	1539.6	1456.3	1221.6	1057.2	813.6

**Table 4:** The dead time of mouse treated with different dose of TTX.

Mice	Weight of Mouse (g)	Dose of Tetrodotoxin (TTX) (µg/Mouse Unit)	Time Until Death (min)
1	19.53	0.20	57
2	20.52	0.20	65
3	21.02	0.20	49
4	19	0.22	28
5	20.81	0.22	30
6	21	0.22	26
7	19.95	0.24	18
8	20.30	0.24	22
9	20.86	0.24	15

**Table 5:** The reduction percentage of TTX by EPS in combination with Cu_2_O reveal by HPLC.

EPS from Selected LAB	TTX Dose (μ/mu)	EPS Dose (μ/mu)	Cuprous Oxide (μ/mu)	Incubation Time (min)	Peak Area	Retention Time (min)	% Reduction
*Leuconostoc mesenteroides*	0.5	250	55	40	30,026	15.038	37.54
	0.5	250	0	40	45,212	15.046	5.96
*Lactobacillus rhamnosus*	0.5	150	55	40	29,899	15.068	37.81
	0.5	150	0	40	34,139	15.064	28.99
*Lactobacillus plantarum*	0.5	100	55	40	44,618	15.063	7.19
	0.5	100	0	40	40,968	15.063	14.78
Cuprous oxide	0.5	0	55	40	31,547	15.083	34.38
Standard TTX	0.5	0	0	40	48,077	15.121	0

**Table 6:** Code level of Plackett–Burman design for *Lactobacillus plantarum*.

Income Variables	Code Levels		
	−1	0	1
EPS (µg/mouse unit) (A)	100	200	300
Cuprous oxide (µg/mouse unit) (B)	10	55	100
Incubated time (min) (C)	20	40	60

**Table 7:** Code level of Plackett–Burman design for *Lactobacillus rhamnosus*.

Income Variables	Code Levels		
	−1	0	1
EPS (µg/mouse unit) (A)	100	150	200
Cuprous oxide (µg/mouse unit) (B)	10	55	100
Incubated time (min) (C)	20	40	60

**Table 8:** Code level of Plackett–Burman design for *Leuconostoc mesenteroides*.

Income Variables	Code Levels		
	−1	0	1
EPS (µg/mouse unit) (A)	100	250	400
Cuprous oxide (µg/mouse unit) (B)	10	55	100
Incubated time (min) (C)	20	40	60

**Table 9:** Plackett–Burman experimental design matrix.

Experiment	Factors		
	A	B	C
1	−1	−1	0
2	−1	1	0
3	1	−1	0
4	1	1	0
5	−1	0	−1
6	−1	0	1
7	1	0	−1
8	1	0	1
9	0	−1	−1
10	0	−1	1
11	0	1	−1
12	0	1	1
13	0	0	0
14	0	0	0
15	0	0	0
